# Pain following thoracoscopic surgery: retrospective analysis between single-incision and three-port video-assisted thoracoscopic surgery

**DOI:** 10.1186/1749-8090-8-153

**Published:** 2013-06-12

**Authors:** Masaya Tamura, Yosuke Shimizu, Yasuo Hashizume

**Affiliations:** 1 Department of Surgery, Fukui Prefectural Hospital, Yotsui 2-8-1, Fukui 910-8526, Japan

**Keywords:** Video assisted thoracoscopic surgery, Postoperative pain, Single incision, Minimally-invasive surgery

## Abstract

**Background:**

The current trend in thoracoscopic surgery is to use fewer ports to decrease postoperative pain, chest wall paresthesia, and duration of hospital stay. In this study we compared the results of our current experience with single-incision thoracoscopic surgery (SITS) and conventional three-port video-assisted thoracoscopic surgery (3P-VATS).

**Methods:**

From October 2011 to August 2012, 37 consecutive patients underwent thoracoscopic surgery. This is a non-randomized retrospective study. Among these patients, 19 (SITS group) were treated using single port method (SITS), whereas 18 (3P-VATS group) were treated using the conventional three-port methods (3P-VATS). The surgical duration, number of resected lesions, duration of chest drainage, duration of hospital stay, inpatient pain scores, and patient satisfaction scores were compared between both groups.

**Results:**

The mean age at surgery, indication, gender, body mass index, and the side involved were similar in both groups. The procedures performed in the SITS group were similar to those performed in the 3P-VATS group. The mean operative time was longer in the SITS group compared with the 3P-VATS group. Duration of postoperative drainage days and hospital stay was shorter in the SITS group compared with the 3P-VATS group, although these differences were not statistically significant. Pain scores on postoperative days 0,1, and 3 were significantly higher in patients who underwent 3P-VATS compared with those who underwent SITS (p = 0.012, 0.039, and 0.037, respectively). The SITS group reported higher patient satisfaction scores than the 3P-VATS group, patients in the 3P-VATS group tended to receive higher total doses of analgesics (NSAIDs) after surgery compared with those in the SITS group, although these differences were not statistically significant.

**Conclusions:**

Our experience demonstrated that SITS decreased postoperative pain and resulted in higher patient satisfaction compared with the conventional three-port VATS. However, a prospective, randomized study is needed to confirm our preliminary findings. To overcome the technological limitations of SITS, the development of new instruments is needed.

## Background

Thoracoscopic surgical techniques have decreased hospital stays, analgesic requirements and postoperative pain compared with the conventional thoracotomy incision [1]. However, more than 50% patients treated with video-assisted thoracoscopic surgery (VATS) report postoperative chest wall paresthesia related to the portal sites [2]. To decrease these complications, conventional VATS has been modified by using fewer and smaller working ports for the surgical procedure.

Thoracic sympathectomy for palmar hyperhidrosis using a single- port technique has been reported and has showed advantages in terms of decreased the duration of hospital stay, rate of postoperative pneumothorax and need for inserting chest drains [3-5]. With the aim of further decreasing VATS invasiveness, Rocco et al. demonstrated the feasibility of performing wedge pulmonary resections through a uniportal VATS approach [6]. With regard to spontaneous pneumothorax, several studies compared single-incision thoracoscopic surgery (SITS) and conventional three-port VATS (3P-VATS) [7-10]. The aim of this study was to compare the results of our current experience with SITS and 3P-VATS.

## Methods

We retrospectively analyzed 37 consecutive patients who underwent thoracoscopic surgery for primary spontaneous pneumothorax, peripheral lung nodules and thymic tumors between October 2011 and August 2012. Among these, 19 patients were treated using SITS, and 18 were treated using the conventional 3P-VATS. For each patient, a retrospective case note review including the following variables was made: age at the time of surgery, gender, body mass index (BMI), surgical time, number of resected lesions, duration of chest drainage (days), duration of hospital stay (days), inpatient pain scores, and patient satisfaction scores.

Maximum pain scores were evaluated using a visual analogue scale (VAS) from 0 to 10. Patient satisfaction was also scored on a scale of 0 to 10. Pain scores were recorded on postoperative days (POD) 0,1,3,7, and 14. Patient satisfaction scores were recorded when patients first visited the outpatient department. Postoperative pain assessment was partially blinded because all scores were recorded by an attending nurse who was unaware of the ongoing study.

After surgery, all patients were followed up for at least 3 months in the outpatient department. The type of surgery was chosen on the basis of patient preference after a written informed consent was obtained. This study was approved by the Institutional Review Board of Fukui Prefectural Hospital.

### Surgical procedures

Besides surgical access, all patients underwent the same surgical procedure, which comprised bullectomy and pleural abrasion, partial lung resection, and thymectomy. The intraoperative analgesic management was standardized in both groups.

### SITS

The SITS technique we used was similar to that described by Rocco ey al [6]. A patient underwent single-lung ventilation and was placed in the lateral decubitus position. A 2.5cm long skin incision was made. The placement of the incision depends on the location of the target area in the chest. The prevalent position was placed in the fifth intercostal space in the mid-axillary line for pneumothorax, and fourth or fifth intercostal space in the anterior position for thymic tumors. A rigid 5-mm 30°video-thoracoscope, a roticulatingendograsper, and an endo-stapler were passed within the same single small incision. Visceral blebs and bullae and the lung were resected using an Endo GIA stapler (Covidien, Norwalk, CT, USA), whereas the thymic tissue was resected using a Liga Sure (Covidien, Norwalk, CT, USA).

### 3P-VATS

An initial 1.5cm long skin incision was made through the previous chest thoracotomy wound (5th or 6th intercostal space). With the lung deflated, two additional 0.5cm skin incisions were made along the anterior-axillary line (4th or 5th intercostal space) and the mid-axillary line (3rd or 4th intercostal space). Lung resection and thymic tumor resection were performed in a manner similar to SITS.

No patients in the SITS group required conversion to 3P-VATS, and no patient in either group required conversion to thoracotomy. Postoperative pain management was standardized and was the same for the two groups. Pain management consisted of routine loxoprofen per os beginning from POD 1, and diclofenac suppository was administered as required. The doses of diclofenac suppositories administered from POD 0 to the day of discharge were compared between the two groups.

### Statistical analysis

All data for continuous variables are expressed as means ± standard deviation. Significant differences between the groups were assessed using Student’s t - tests for continuous variables, and χ^2^-tests for categorical variables. Statistical analyses were performed using the SAS software package (SAS Institute, Inc, Cary, NC). A p-value of <0.05 was considered significant.

## Results

The mean age at the time of surgery, indications, genders, BMI indices, and the sides involved were similar for both groups of patients (Table 1). The procedures performed in the SITS group were also similar to those performed in the 3P-VATS group. The patients’perioperative outcomes are listed in Table 2.

**Table 1:** **Clinical characteristics of patients who underwent SITS and 3P**- **VATS**

	**SITS (n**=**19)**	**3P**-**VATS (p**=**18)**	**p-value**
Indications			0.691
Pneumothorax	10 (52.6%)	10 (55.6%)	
Lung nodule	5 (26.3%)	6 (33.3)	
Thymictumor	4 (21.1%)	2 (11.1%)	
Age (years)	44.6±5.4	43.4±5.5	0.878
Gender			0.801
Male	13 (68.4%)	13 (72.2%)	
Female	6 (31.6%)	5 (27.8%)	
BMI (kg/m^2^)	21.3±0.87	21.4±0.90	0.966
Side involved			0.861
Right	11 (57.9%)	10 (55.6%)	
Left	4 (42.1%)	8 (44.4%)	

*SITS* Single incinsionthoracoscopic surgery, *3P-VATS* 3-port video assisted thoracoscopic surgery.

**Table 2:** **Surgical characteristics of patients in the SITS and 3P**- **VATS groups**

	**SITS (n**=**19)**	**3P**-**VATS (p**=**18)**	**p-value**
Operation time (min)	60.5±3.1	58.8±3.2	0.692
Number of resected lesions		0.101	
1	14 (73.7%)	13 (72.2%)	
2	4 (21.0%)	2 (11.1%)	
3	0 (0%)	3 (16.7%)	
4	1 (5.3%)	0 (0%)	
Post operative drainage (days)	1.11±0.09	1.22±0.09	0.349
Post operation hospital stays (days)	3.85±0.27	4.33±0.28	0.271
Wound infection	0 (0%)	1 (5.6%)	0.663

*SITS* Single incinsionthoracoscopic surgery, *3P-VATS* 3-port video assisted thoracoscopic surgery.

**Figure 1:**
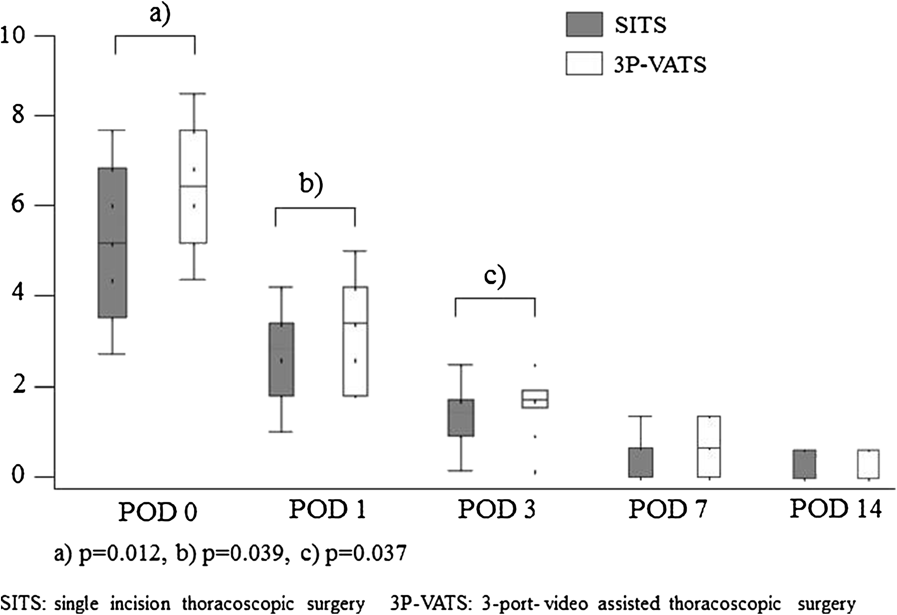
**Postoperative pain scores on a visual analogue scale according to the number of ports as a function of time after thoracoscopic surgery.** Pain scores on postoperative days 0,1, and 3 were higher for patients who underwent three-port VATS than for those who underwent SITS (p = 0.012, 0.039 and 0.037, respectively).

The mean operative time was longer for the SITS group. The duration of postoperative drainage and hospital stay were shorter for the SITS group compared with the 3P-VATS group, although these differences were not statistically significant. There were no intraoperative complications, although one patient who underwent 3P-VATS developed a wound infection. The mean postoperative pain scores, patients’satisfaction scores, and analgesic doses are listed in Table 3. In the SITS group, the VAS scores on POD 0, 1, 3 were 4.95 ± 0.38, 2.74 ± 0.34, and 1.32 ± 0.20, respectively. These VAS scores in the 3P-VATS group, were 6.44 ± 0.39, 3.78 ± 0.35, and 1.94 ± 0.21, respectively. Pain scores on POD 0,1, and 3 were significantly higher for patients who underwent 3P-VATS than for those who underwent SITS (p = 0.012, 0.039, and 0.037, respectively) (Figure 1). The SITS group reported higher patient satisfaction scores than the 3P-VATS group, although these were not significantly different (p = 0.078). Patients in the 3P-VATS group tended to receive higher total doses of analgesics (NSAIDs) after surgery compared with the SITS group, but this difference was not statistically significant (p = 0.119) (Table 3). No patients showed side effect of analgesic. One patient of pneumothorax in the 3P-VATS group revealed recurrence 2 months after surgery.

**Table 3:** **Visual analog scale scores and patient satisfaction scores for SITS and 3P**- **VATS patients**

	**SITS (n**=**19)**	**3P**-**VATS (p**=**18)**	**p-value**
Visual analog scale (VAS) score			
POD 0	4.95±0.38	6.44±0.39	0.012
POD 1	2.74±0.34	3.78±0.35	0.39
POD 3	1.32±0.20	1.94±0.21	0.37
POD 7	0.42±0.18	0.83±0.18	0.116
POD 14	0.26±0.11	0.39±	0.428
Satisfaction scores	8.95±0.24	8.33±0.24	0.078
Analgesic doses*	0.89±0.24	1.44±0.94	0.119

*The number of diclofenac suppository taken from postoperative day 0 to discharge was compared between two groups. *SITS* Single incinsionthoracoscopic surgery, *3P-VATS* 3-port video assisted thoracoscopic surgery.

## Discussion

This retrospective study demonstrated the advantages of the SITS technique over conventional 3P-VATS are primarily related to postoperative pain, especially during the acute phase. And no difference of complication and prognosis could be seen between the two approach.

During the last two decades VATS has been performed with increasing frequency for treating lung cancer. Although this surgery may be performed using one or two ports, most of surgeons use three incisions. For the first time, Yamamoto et al. in 1998 [11] reported the use of a single-incision VATS for six patients with pneumothorax. Later, Rocco et al. in 2004 [6] reported that a similar technique was an effective approach for safely performing wedge resections for pulmonary lesions.

Because only one intercostal space is involved, the potential advantages of SITS are expected to include less postoperative pain, fewer postoperative drainage days, shorter hospital stays, and cosmetic advantages compared with the conventional 3P-VATS. Some authors have reported less postoperative pain and less paresthesis in patients who underwent minor procedures through a single-port approach compared with the classical three-port approach [7]. Jutley et al. [8] reported less postoperative pain and a lower incidence of residual paresthesia in patients who underwent a single-incision VATS for spontaneous pneumothorax.

In the present study, there were no significant differences between the two groups of patients in terms of the duration of surgery, postoperative drainage, or hospital stays. However, SITS resulted in less postoperative pain. Moreover, the SITS group reported higher satisfaction scores although not statistically significant. This may have been attributed to decreased postoperative pain and cosmetic superiority.

With regard to single-port thoracoscopic surgery, a technological problem is obvious. SITS is not a naturally ergonomic procedure, because the traditional thoracoscopic principles of triangulation are lost. In addition, positioning multiple devices poses a problem because they are passed through a single small incision in the chest. Instruments often interfere with each other, not only within the pleural space, but also extrapleurally, where attachments like a camera light lead often impede movements.

**Figure 2:**
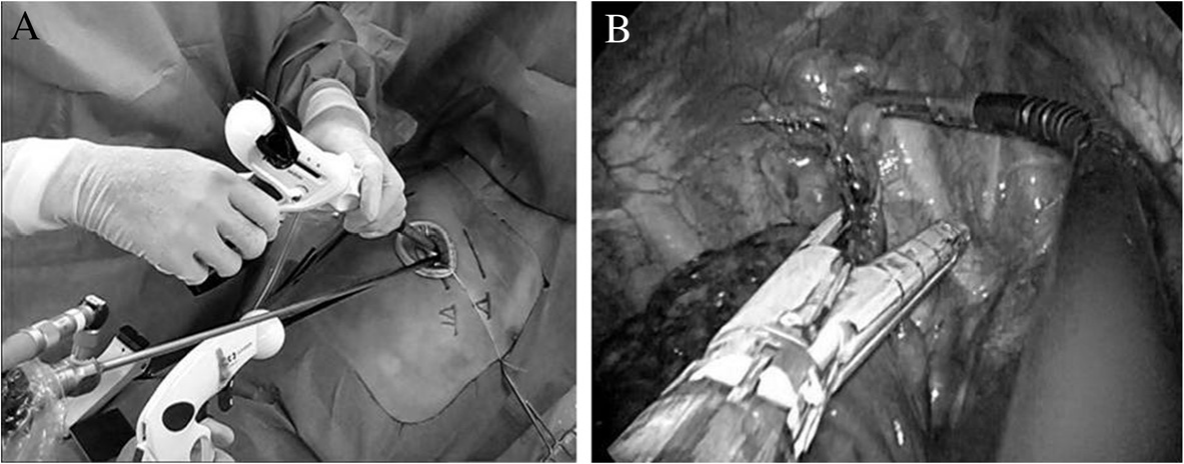
External and intraoperative views during SITS.

To overcome these limitations, the development of new instruments is needed. Increasing the length of the camera shaft would allow an assistant to stand comfortably with his/her hands away from those of the operating surgeon (Figure 2A). A roticulating end grasper has aided in achieving triangulation and using these devices with good results (Figure 2B). Nevertheless, thoracic surgeons still use instruments that are adapted from conventional thoracic surgeries, for example, a single-incision laparoscopic surgery port (SILS port) [9]. Therefore, developing a proper thoracic single port is mandatory. In future, we believe that these issues may be resolved with new, inline instruments, that will avoid such interferences.

A limitation of this investigation is that our data were retrospectively collected in a small samples, it was not a randomized double blind study. We appreciate the potential self-selection bias in our study as the patients had chosen the type of surgery. However, we included various procedures, which were performed in the same manner in both the SITS and 3P-VATS groups.

Randomized controlled trials and many aspects need further research before SITS is wildly used in clinical practice.

## Conclusions

Our experience demonstrated that SITS decreased postoperative pain and resulted in higher patient satisfaction compared with the conventional three-port VATS.

## Abbreviations

SITS: Single-incision thoracoscopic surgery; VAS: Visual analog scale; VATS: Video- assist thoracoscopic surgery.

## Competing interests

The authors declare that they have no competing interests.

## Authors’ contributions

YS analyzed and interpreted the patient data. MT performed the literature review, and was a major contributor in writing the manuscript. YH performed the final editing of the manuscript. All authors read and approved the final manuscript.

## References

[B1] Hazelrigg SR Magee MJ Boley TM Yim APC, Hazelrigg SR, Izzat MB, Landreneau RJ, Mack MJ, Naunheim KS Spontaneous pneumothorax Minimal access cardiothoracic surgery 2000 Philadelphia, PA: Saunders 73 79

[B2] Sihoe AD Au SS Cheung ML Chow IK Chu KM Law CY Wan M Yim AP Incidence of chest wall paraesthesia after video-assisted surgery surgery for primary spontaneous pneumothorax Eur J CardiovascSurg 2004 24 1054 1058 10.1016/j.ejcts.2004.02.018 15145009

[B3] Lardionois D Ris HB Minimally invasive video-endoscopic sympathectomy by use of a transaxillary single port approach Eur J CardiothoracSurg 2002 21 1 67 70 10.1016/S1010-7940(01)01042-9 11788259

[B4] Chen YB Ye W Yang WT Shi L Guo XF Xu ZH Qian YY Uniportal versus biportal video-assisted thoracoscopicsympathectomy for palmar hyperhidrosis Chin Med J 2009 122 13 1525 1528 19719942

[B5] Murphy MO Gohsh J Khwaja N Murray D Halka AT Carter A Turner NJ Walker MG Upper dorsal endoscopic thoracic sympathectomy: a comparison of one-and two ablation techniques Eur J CardiothoracSurg 2006 30 223 227 10.1016/j.ejcts.2006.04.016 16829101

[B6] Rocco G Martin-Ucar A Passera E Uniportal VATS wedge pulmonary resections Ann ThoracSurg 2004 77 726 728 10.1016/S0003-4975(03)01219-0 14759479

[B7] Salati M Brunelli A Xiume F Refai M Sciarra V Soccetti A Sabbatini A Uniportal video-assisted thoracic surgery for spontaneous pneumothorax: clinical and economic analysis in comparison to the traditional approach InteractCardiovascThoracSurg 2008 7 63 66 10.1510/icvts.2007.165712 17984169

[B8] Jutley RS Khalil MW Rocco G Uniportalvs standard three-port VATS technique for spontaneous pneumothorax: comparison of post-operative pain and residual paraesthesia Eur J CardiothoracSurg 2005 28 43 46 10.1016/j.ejcts.2005.02.039 15927479

[B9] Berlanga L Gigirey O Uniportal video-assisted thoracic surgery for primary spontaneous pneumothorax using a single-incision laparoscopic surgery port: a feasible and safe procedure SurgEndosc 2011 25 2044 2047 10.1007/s00464-010-1470-7 21136111

[B10] Chen PR Chen CK Lin YS Huang HC Tsai JS Chen CY Fang HY Single-incision thoracoscopic surgery for primary spontaneous pneumothorax J CardiothoracSurg 2011 21 6 58 10.1186/1749-8090-6-58 PMC3094379 21507268

[B11] Yamamoto H Okada M Tanada M Matsuoka H Sakata K Kawamura M Video-assisted thoracic surgery through a single skin incision Arch Surg 1998 133 145 14 10.1001/archsurg.133.2.145 9484725

